# Recovery of a human natural antibody against the noncollagenous-1 domain of type IV collagen using humanized models

**DOI:** 10.1186/s12967-015-0539-4

**Published:** 2015-06-06

**Authors:** Inge M Worni-Schudel, Amy G Clark, Tiffany Chien, Kwan-Ki Hwang, Benny J Chen, Mary H Foster

**Affiliations:** Department of Medicine, Duke University Medical Center, Durham, NC USA; Durham VA Medical Center, Durham, NC USA; Duke Human Vaccine Institute, Duke University Medical Center, Durham, NC USA; Duke Cancer Institute, Duke University Medical Center, Durham, NC USA

## Abstract

**Background:**

Anti-glomerular basement membrane nephritis and Goodpasture syndrome result from autoantibody (Ab)-mediated destruction of kidney and lung. Ab target the noncollagenous 1 (NC1) domain of alpha3(IV) collagen, but little is known about Ab origins or structure. This ignorance is due in part to the inability to recover monoclonal Ab by transformation of patients’ blood cells. The aim of this study was to assess the suitability of two humanized models for this purpose.

**Methods:**

NOD-scid-gamma immunodeficient mice were engrafted either with human CD34+ hematopoietic stem cells (HSC) (Hu-HSC mice) and immunized with alpha3(IV)NC1 collagen containing the Goodpasture epitopes or with nephritis patients’ peripheral blood leukocytes (PBL) (Hu-PBL mice). After in vivo immune cell development and/or expansion, recovered human B cells were Epstein Barr virus (EBV)-transformed, screened for antigen (Ag) binding, electrofused with a mouse–human heterohybridoma, subcloned, and human Ab RNA sequenced by PCR after reverse transcription to cDNA. Flow cytometry was used to assess human B cell markers and differentiation in Hu-PBL mice.

**Results:**

Sequence analysis of a human Ab derived from an immunized Hu-HSC mouse and reactive with alpha3(IV)NC1 collagen reveals that it is encoded by unmutated heavy and light chain genes. The heavy chain complementarity determining region 3, a major determinant of Ag binding, contains uncommon motifs, including an N-region somatically-introduced highly hydrophobic tetrapeptide and dual cysteines encoded by a uniquely human IGHD2-2 Ab gene segment that lacks a murine counterpart. Comparison of human and mouse autoantibodies suggests that structurally similar murine Ab may arise by convergent selection. In contrast to the Hu-HSC model, transformed human B cells are rarely recovered from Hu-PBL mice, in which human B cells terminally differentiate and lose expression of EBV receptor CD21, thus precluding their transformation and recovery.

**Conclusions:**

Hu-HSC mice reveal that potentially pathogenic B cells bearing unmutated Ig receptors reactive with the NC1 domain on alpha3(IV) collagen can be generated in, and not purged from, the human preimmune repertoire. Uniquely human gene elements are recruited to generate the antigen binding site in at least a subset of these autoantibodies, indicating that humanized models may provide insights inaccessible using conventional mouse models.

**Electronic supplementary material:**

The online version of this article (doi:10.1186/s12967-015-0539-4) contains supplementary material, which is available to authorized users.

## Background

Anti-glomerular basement membrane (anti-GBM) glomerulonephritis is a human autoimmune disease that typically presents with acute kidney injury or hematuria. A subset of patients develops autoimmune lung injury, a combination referred to as Goodpasture’s syndrome. In its most severe manifestation, rapidly progressive glomerulonephritis and alveolar hemorrhage can lead to organ failure and death. Hallmarks of the disease are the presence of linear antibody deposits along the basement membranes of renal glomeruli and circulating Ab that bind the noncollagenous-1 (NC1) domain of the alpha3 chain of type IV collagen [α3(IV)NC1], the major target antigen in affected basement membranes [1, 2]. Direct pathogenicity was suggested by rapid recurrence of disease in renal allografts established in the presence of persistent circulating anti-GBM Ab [3], and confirmed by passive transfer of patients’ kidney eluate Ab into squirrel monkeys [4].

Anti-GBM glomerulonephritis is considered the prototype human autoimmune nephritis because the target antigen is well characterized [5]. Yet despite considerable advances in defining antigenic epitopes, little is known about the origins and molecular basis of human anti-GBM Ab or their regulatory control. On the one hand, pathogenic patient Ab are high affinity and target a limited number of epitopes [6], suggesting an antigen driven immune response. Conversely, low titers of anti-GBM IgG that recognize the same epitopes as patient anti-GBM IgG can be identified in serum of healthy individuals using sensitive techniques [7, 8], suggesting their presence in the healthy immune repertoire.

Barriers to further progress in this field include a paucity of suitable model systems and an inability to recover human monoclonal Ab (mAb) from patients. Whereas experimental anti-GBM glomerulonephritis can be induced by immunization under some conditions [9–11], patient-derived Goodpasture Ab bind poorly to native (untreated) mouse kidney and to undissociated rat kidney alpha3(IV)NC1 hexamers [12, 13]. Injection of human Goodpasture Ab into mice does not induce glomerulonephritis, a finding attributed in part to extensive alpha3(IV)NC1 hexamer crosslinking in rodents that minimizes pathogenic epitope exposure and prevents IgG binding to Ag in vivo [12]. Nonetheless, rodents express Goodpasture antigen [12], and mice bearing humanized immune components should be useful for generating and studying origins and regulation of human anti-GBM Ab in vivo. Herein we assess the suitability of humanized NSG mice for this purpose. We find that human anti-alpha3(IV)NC1 collagen autoantibodies can be recovered from immunized Hu-HSC mice, and that the antigen binding site in at least a subset of these autoantibodies is generated using uniquely human Ig gene elements. In contrast, utility of the Hu-PBL model for human monoclonal autoantibody recovery using EBV transformation is limited by extensive human B cell terminal differentiation with loss of CD21 expression.

## Methods

### Human blood cell isolations

De-identified human cord blood was obtained from the Carolinas Cord Blood Bank. CD34+ HSC were isolated by density gradient (Ficoll-Paque^™^ PLUS, GE Healthcare, Chalfont St. Giles, UK) followed by magnetic separation (CD34 MultiSort Kit, Miltenyi Biotec, Auburn, CA, USA). Blood was collected from two subjects with anti-GBM glomerulonephritis within 6 days of renal biopsy and from healthy donors, after informed consent as approved by the Institutional Review Board of Duke University, and PBL isolated by density gradient (Ficoll-Paque^™^ PLUS, GE Healthcare).

### Animals

Female NSG mice were purchased from The Jackson Laboratory (Bar Harbor, ME, USA) and the Duke Cancer Center Isolation Facility Breeding Core and housed under specific pathogen free conditions. For Hu-HSC mice, 14-week-old NSG recipients were intravenously injected with 5 × 10^5^ CD34+ HSC per mouse 4 h after irradiation (225 cGy, X-RAD 320, Precision X-ray, North Branford, CT) [14, 15], followed by indefinite oral sulphamethoxazole–trimethoprim administered in water. For Hu-PBL mice, 7–15 week old NSG recipients received 6–10 × 10^6^ PBL per mouse [15]. All studies were approved by the Duke University Animal Care and Use Committee and conform to institutional standards.

### Immunization and mouse studies

Fourteen weeks after HSC injection, Hu-HSC mice were immunized with 25 μg bovine alpha3(IV)NC1 collagen (Eurodiagnostica, Malmo, Sweden) emulsified in PBS and Freund’s Complete Adjuvant, followed by boosts at week 17 and 20 with antigen in Freund’s Incomplete Adjuvant. Tissue was harvested at week 23 post-engraftment. For urine collection, mice were housed for 18–24 h in mouse metabolic cages with access to food and water. Mouse serum and urine creatinine were measured by the University of North Carolina Animal Chemical Chemistry and Gene Expression Laboratories Core Facility using an automated Chemical Analyzer VT350 (Ortho Clinical Diagnostics, Rochester, NY, USA), and urine albumin was measured using a mouse Albuwell quantitation kit (Exocell, Philadelphia, PA, USA). Kidneys were fixed in 10% formalin then embedded in paraffin, or snap-frozen in OCT. All sections were cut and stained by the Duke Surgical Sciences Histopathology Lab Core Facility. For direct immunofluorescence, sections were stained with goat-anti-human IgM or IgG-Alexa Fluor488 antibodies (Molecular Probes, Eugene, OR, USA), and images acquired using NIS-Elements software (Nikon USA).

### Flow cytometry

Single-cell suspensions of human PBL and mouse cells were analyzed by flow cytometry as described [16], using fluorescent-conjugated antibodies (Becton–Dickinson-Pharmingen, San Jose, CA, USA). Cells were analyzed on a FACSCalibur or BD CANTO apparatus (Becton–Dickinson-Pharmingen) and results analyzed using FlowJo (Treestar, Ashland, OR, USA), with live cells gated on forward and side scatter. Chimerism, or the level of human lymphocyte engraftment, in spleen was calculated as follows: %Chimerism = (%human CD45+ cells × 100)/(%human CD45+ cells + %mouse CD45+ cells).

### Cell culture and EBV transformation

Harvested cells were cultured in bulk overnight at 37°C in 5% CO_2_ in complete medium: RPMI-1640 (Sigma-Aldrich, St. Louis, MO, USA) supplemented with 15% fetal bovine serum (Gibco/Life Technologies, Grand Island, NY, USA), 2 mM l-glutamine (Sigma-Aldrich) and 1 mM sodium pyruvate, 1% nonessential amino acids (a.a.), 10 mM Hepes buffer, and 100 U/ml penicillin and 100 µg/ml streptomycin (Gibco/Life Technologies), containing 1 μg/ml Cyclosporin A (Sigma-Aldrich) for T cell inhibition, 2.5 μg/ml CpG ODN2006 (InvivoGen, San Diego, CA, USA) for TLR stimulation, 5 μM checkpoint 2 (CHK2) kinase inhibitor (Calbiochem/EMD Chemicals, Gibbstown, NJ, USA), and 1 ml EBV B95-8 virus suspension per 10^7^ cells (American Type Culture Collection, Rockville, MD, USA). Cells were then seeded in 96-well plates, and refed at day 7 with medium lacking cyclosporin A and then once weekly with medium also lacking CpG oligonucleotides [17].

### Hybridoma generation

HMMA2.5 mouse–human chimera myeloma cells (gift of Lisa Cavacini, Beth Israel Deaconess Medical Center, Boston) [18] and EBV-stimulated B cells were washed three times with isoosmolar electrofusion medium (Eppendorf, Hauppauge, New York, USA) then electrofused with a PA-4000/PA-101S apparatus, as described [19]. After overnight incubation in 96-well plates, medium was supplemented with 100 μM hypoxanthine, 0.4 μM aminopterin, 16 μM thymidine, and 0.5 μM ouabain. Antigen-reactive lines were subcloned using a standard limiting dilution method.

### Enzyme linked immunosorbent assay (ELISA)

Human immunoglobulin (Ig) and anti-alpha3(IV)NC1 collagen Ab in serum and fusion cell supernatants were detected by ELISA, as described [20], using the following reagents: Human IgG and IgM standards (Thermo Scientific, Waltham, MA, USA), goat-anti-human Ig or goat-anti-human-kappa capture Ig, and alkaline-phosphatase conjugated goat anti-human Ig, anti-human IgG, and anti-human IgM (Southern Biotech, Birmingham, AL, USA). Results for binding to antigen [bovine alpha3(IV)NC1 collagen (Eurodiagnostica)] were recorded as OD on antigen minus OD on wells coated with diluent only (6 M guanidine-HCl, Sigma-Aldrich), using as controls mouse anti-alpha3(IV)NC1 collagen IgG (Mab3) and Goodpasture patient serum (Eurodiagnostica).

### Analysis of human anti-alpha3(IV)NC1 mAb variable region genes

RNA was isolated from the 2D6 heterohybridoma cells expressing the anti-alpha3(IV)NC1 collagen IgM/lambda Ig using the RNeasy mini kit (Qiagen, Valencia, CA, USA), converted to cDNA using the High Capacity cDNA Reverse Transcription Kit (Life Technologies, Grand Island, NY, USA), and productively rearranged Ig heavy and light chain variable region genes amplified by PCR, using published panels of 5′ primers for human VH (VH1-Ext–VH6-Ext) and Vlambda (Vlambda1-Ext–Vlambda10-Ext) subgroups [21], and a 3′ primer for the IgM and lambda constant region (5′-CCGACGGGGAATTCTCACAG-3′ and 5′-AGGCCACTGTCACAGCT-3′, respectively). PCR products were purified using the QIAquick PCR Purification Kit (Qiagen) and sequence determined by the Duke University DNA Analysis facility. Sequences were analyzed using the International ImMunoGeneTics Information System (IMGT) public Ig gene database (http://www.imgt.org) with the IMGT/VQUEST tool [22] integrated with IMGT/JunctionAnalysis [23]. Sequence comparisons were also performed using the BLAST software and databases (blast.ncbi.nlm.nih.gov/Blast.cgi).

### Statistical analyses

Data are shown as mean ± standard deviation (SD) or median (range), as indicated. Comparisons between two groups were performed using a nonparametric Wilcoxon rank-sum test and Stata (College Station, TX, USA) and JMP (SAS Institute, Cary, NC, USA) software, with *p* < 0.05 considered significant.

## Results

### Recovery of human B cells and anti-α3(IV)NC1 collagen Ab from immunized Hu-HSC mice

**Figure 1:**
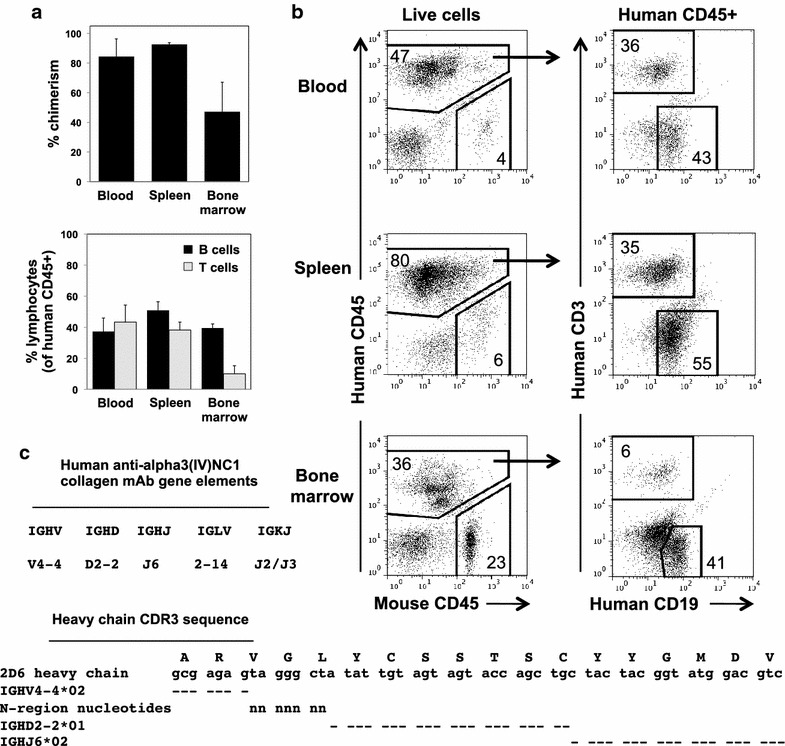
Human anti-alpha3(IV)NC1 collagen mAb derived from an immunized Hu-HSC mouse. **a** Human chimerism and lymphocyte subsets from two immunized Hu-HSC mice 23 weeks after engraftment, calculated as described in “Methods”. Shown are mean (±SD). **b** Representative flow cytometric analysis, with cell gates indicated at the *top* and percentage of cells indicated within each marker. **c** Gene segments encoding heavy and light chain variable (V) regions and sequence of the heavy chain CDR3 (delineated according to the IMGT unique numbering for V-DOMAIN [43]), compared to that of the closest corresponding germline V region gene segment alleles, identified in the IMGT/V-QUEST reference database. *Dashes* represent sequence identity with mAb.

A human lymphoid system was established in vivo by injection of conditioned adult NSG mice with CD34+ human HSC. Recipients with good engraftment were immunized with bovine alpha3(IV)NC1 collagen at 14, 17 and 20 weeks post-engraftment. At tissue harvest at 23 weeks, mean chimerism ranged from 33 to 93%, with highest levels in spleen (Figure 1a, b). Of human CD45+ cells in spleen, B cells and T cells constituted mean 50.85 and 38.2%, respectively (Figure 1a, b). Plasma contained mean 87.9 ± 38.3 µg/ml human IgM and 5.7 ± 2.3 µg/ml human IgG, and low levels of alpha3(IV)NC1 collagen-reactive human IgM (mean OD405 0.107 ± 0.030, Hu-HSC mice, compared to 0.002 ± 0.002 in unengrafted NSG mice). Ag-reactive IgG was not detected (mean OD405 0.010 ± 0.000).

We did not find evidence of renal injury in the immunized Hu-HSC mice, based on plasma creatinine at the time of tissue harvest (mean 0.1 ± 0.0 mg/dl) and absence of glomerular crescents on renal histopathological examination (not shown). Urine albumin excretion was higher in Hu-HSC mice at 2.5 months after immunization (mean 98.6 ± 18.6 µg alb/mg Cr, compared to 46.4 ± 6.0 µg alb/mg Cr prior to immunization). On direct immunofluorescence examination of kidneys, scattered deposits of human IgG and IgM were detected in glomeruli, with faint linear capillary wall human IgM staining in some glomeruli (not shown), whereas no human Ig was detected in kidneys from control unengrafted NSG mice.

One week after splenocyte culture with CpG oligonucleotides and EBV (240 wells, 70,000–75,000 human B cells/well), human Ig was detected in 100% of tested wells (n = 60). All contained human IgM (>10 µg/ml) and 62% contained human IgG, at low levels (<0.2 µg/ml). At 1 month, 49.6% of wells had cell clusters, and 35.8% contained Ag-reactive Ig; Ig in several wells also bound diluent-coated wells. A cell line producing Ag-reactive Ig and maintaining favorable growth characteristics was expanded, electrofused, and subcloned by limiting dilution. The resulting hybridoma produced a human anti-alpha3(IV)NC1 collagen IgM, lambda mAb, termed 2D6.

### Sequence analysis of human anti-α3(IV)NC1 collagen mAb 2D6

The rearranged variable region genes encoding 2D6 were determined by sequence analysis after RT-PCR of cDNA. For each panel of primers, a single PCR band of correct size and a single sequence were identified, consistent with monoclonality. The gene segments and nucleotide and deduced a.a. sequences are shown in Figure 1c (and Additional files 1, 2: Figures S1, S2), and available in the DDBJ/EMBL/GenBank nucleotide databases under the accession numbers KP261837 and KP261838.

The mAb heavy chain (HC) is encoded by an IGHV4-4 gene with 100% identity to an unmutated IGHV4-4*02 allele. Review of the databases reveals that this unmutated gene is used by multiple human Ig reactive with self and foreign Ag (not shown). The HC CDR3 is encoded by 7 N1-region nontemplated (somatically generated) nucleotides and by 21 nucleotides contributed by an unmutated IGHD2-2 gene, allele IGHD2-2*01, translated in reading frame 2 (Figure 1c). Additional nucleotides at either end of HC CDR3 can be accounted for by the unmutated IGHV4-4*02 allele and an unmutated IGHJ6 gene, allele IGHJ6*02, that also encodes framework (FR) 4.

The 2D6 light chain uses an unmutated IGLV2-14 gene (also known as 2a2 or 1-4), with 100% identity to the IGLV2-14*03 allele, noting that current databases are missing the first 8 FR1 codons of this allele. 2D6 uses an unmutated IGLJ2 or IGLJ3 gene, with 100% match to alleles IGLJ2*01 and IGLJ3*01, which have identical nucleotide sequences in the expressed region.

### Human B cells and antibodies in Hu-PBL mice established with nephritis patient leukocytes

**Figure 2:**
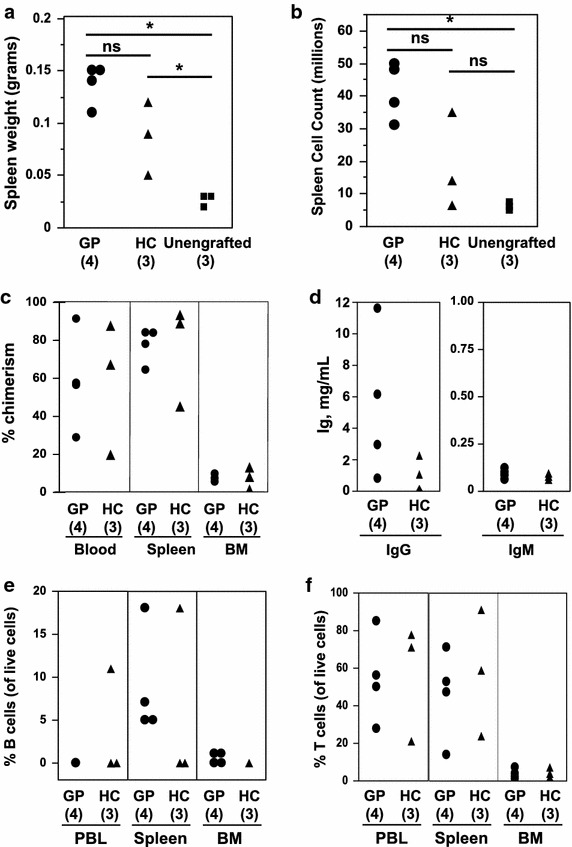
Engraftment of NSG mice with PBL from two patients with anti-GBM glomerulonephritis. NSG mice were injected with PBL from glomerulonephritis patients (GP) (*circles*) or from a healthy control subject (HC) (*triangles*), or were unengrafted (*squares*). Shown are spleen weight and cell count (**a**, **b**), % chimerism (**c**), serum immunoglobulin levels (**d**), and % human B cells (**e**) and T cells (**f**) in tissue compartments. *Each symbol* represents an individual Hu-PBL mouse. Chimerism was calculated as described in “Methods”. *PBL* peripheral blood leukocytes, *BM* bone marrow; *p < 0.05.

To assess the suitability of Hu-PBL mice as a reservoir to expand and recover human anti-GBM B cells from patients, NSG mice were injected with PBL isolated from two patients (GP01 and GP02) with active anti-GBM glomerulonephritis and detectable circulating anti-alpha3(IV)NC1 antibodies (n = 4 mice, two each with PBL from a patient) or from a healthy donor (n = 3 mice). At organ harvest 3–6 weeks later, cell counts and chimerism were similar in mice injected with patient versus healthy donor cells (Figure 2), with chimerism highest in the spleen (77.58% ± 9.24 vs 75.48% ± 26.46, mean ± SD, for patient vs healthy donor Hu-PBL mice, p = NS). The majority of human leukocytes in Hu-PBL mice were CD3+ T cells, whereas B cells constituted a small proportion of cells (Figure 2), consistent with the normal low frequency of B cells in human PBL.

Hu-PBL mice nonetheless had substantial blood levels of human IgG and IgM (Figure 2), indicating either transfer of activated B cells or xenoactivation of resting B cells. Mean plasma concentrations were 5,374.80 ± 4,709.71 and 1,164.56 ± 1,086.03 μg/ml, human IgG, and 92.96 ± 25.45 and 77.44 ± 16.60 μg/ml, human IgM, for nephritis- and healthy donor-derived Hu-PBL mice, respectively. Only low level human Ig activity against alpha3(IV)NC1 collagen was detected in Hu-PBL mouse plasma (at 1:50 dilution, mean ± SD, OD 0.057 ± 0.025, n = 4, for patient-derived Hu-PBL, and OD 0.047 ± 0.046, n = 3, for healthy donor-derived Hu-PBL; with reference OD −0.004 ± 0.004, n = 2, unengrafted NSG plasma and OD 1.099 for control patient serum at 1/300 dilution).

In contrast to Hu-HSC mice, few human B cell lines were recovered from Hu-PBL mice. At 1 month, no clones were visible in EBV-infected cultures derived from glomerulonephritis donor GP01 Hu-PBL mice. Cultures from glomerulonephritis donor GP02 Hu-PBL mice were observed for 2 months, at which time proliferation consistent with EBV transformation was observed in a small subset of wells (55 of 1,728, 3.2%) plated with splenocytes (mean ± SD 4,738 ± 3,688 human B cells/well).

### Loss of human CD21 in Hu-PBL

**Figure 3:**
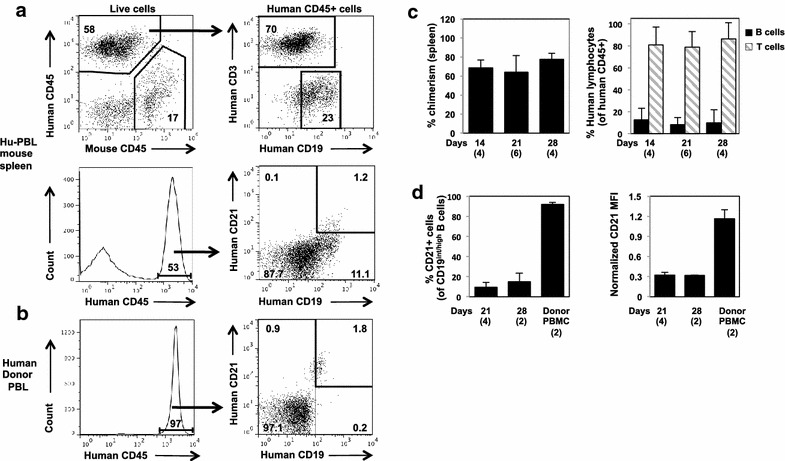
CD21 expression on human B cells from Hu-PBL mice engrafted with healthy donor cells. **a** and **b** Representative flow cytometry; Hu-PBL mouse is 28 days post engraftment. Gates and percentages of gated cells are indicated. **c** Spleen chimerism, and human lymphocyte subsets; day after engraftment as indicated. **d** CD21 expression, gated on human B cells with intermediate-to-high CD19 expression. MFI, mean fluorescence intensity, normalized to the human donor PBL control in each experiment. Shown are mean and SD; number of subjects is shown in parentheses; *p < 0.05.

**Figure 4:**
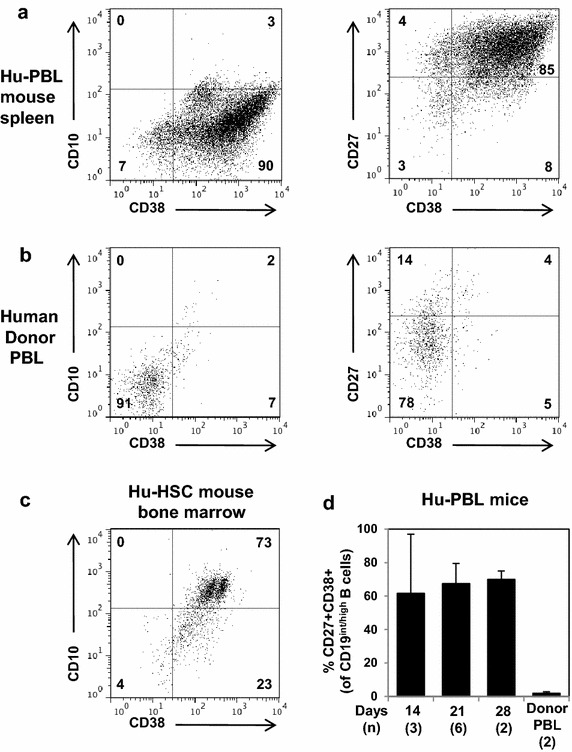
Humanized mouse and healthy human donor B cell subset analysis. **a**–**c** Representative flow cytometric analysis after gating on human CD45+ CD19+ live cells. The Hu-PBL (**a**) and alpha3(IV)NC1 collagen-immunized Hu-HSC (**c**) mice are 14 and 162 days post engraftment, respectively. Percentage of gated cells are indicated. **d** %CD27+ CD38+ expression on intermediate-to-high CD19+ B cells from splenocytes of Hu-PBL mice engrafted with healthy donor PBL, gated as shown in (**a**).

Failure of EBV to capture the human B cells from Hu-PBL mice suggested that expression of complement receptor CD21, critical for EBV attachment and infection, was altered on mature human B cells after adoptive transfer. To explore this possibility, we generated Hu-PBL mice from two additional healthy donors and analyzed CD21 expression and B-cell subsets at 14, 21, and 28 days (n = 4–6 mice per time point) (Figures 3, 4). Consistent with their low frequency in healthy human blood (Figure 3b), B cells constituted 0–25% (mean 9.96% ± 8.79, n = 14) of Hu-PBL mouse engrafted human leukocytes (Figure 3c). Whereas CD21 was expressed on the vast majority of healthy donor PBL B cells, a minority of human B cells from Hu-PBL mice expressed CD21 (Figure 3a, d), as quantified for B cells with intermediate-to-high CD19 expression, comparable to that of injected donor B cells (Figure 3b, d). At 21–28 days after cell injection, only mean 11.33% ± 6.02 of CD19^int/high^ human B cells recovered from spleens of Hu-PBL mice express CD21, and CD21 surface density was 68% lower on these cells (normalized mean fluorescent intensity, or MFI, 0.32 ± 0.03, n = 6), compared to concurrently tested CD19+ CD21+ human control blood cells (normalized MFI 1.0). The true frequency and density of CD21 expression on Hu-PBL mouse B cells is even lower; CD19-low B cells in Hu-PBL mouse spleen are CD21-negative, but are excluded from analysis due to overlap with CD3+ cells in the gating algorithm (Figure 3a).

Subset analysis indicates that the majority of human B cells recovered from Hu-PBL mouse spleens are CD10-negative (Figure 4a), consistent with mature B cells and similar to the vast majority of donor blood B cells (Figure 4b) and in contrast to immature human CD19+ B cells developing in bone marrow of engrafted Hu-HSC mice (Figure 4c). The majority of Hu-PBL human B cells are CD27+ CD38+ (Figure 4a, d), suggesting terminal differentiation to plasmablasts and plasma cells.

## Discussion

We assessed two humanized models for their suitability for in vivo study of anti-GBM immune responses and recovery of human anti-alpha3(IV)NC1 collagen B cells and mAb. We found that a subset of Ag-reactive B cells from immunized Hu-HSC mice are amenable to EBV transformation, and that this permitted successful fusion and generation of an Ag-reactive B cell clone. Sequence analysis revealed an Ag-reactive human mAb encoded by unmutated variable region genes, suggesting that potentially pathogenic anti-alpha3(IV)NC1 collagen B cells, or their precursors, are generated in, and not purged from, the human preimmune repertoire and contribute to the natural Ab pool. Sequence analysis also revealed use of rare and uniquely human elements in the mAb HC Ag binding region, further suggesting that the humanized model can unmask disease-relevant features not accessible in a non-humanized mouse model. In contrast, human anti-GBM B cell lines were not recovered from Hu-PBL mice engrafted with patient PBL, at least in part due to loss of the EBV receptor, CD21, from human B cells as they terminally differentiate in the NSG host. Thus, humanized models provide insight into origins of pathogenic human anti-collagen autoimmunity, while posing unique opportunities and challenges.

The antibody HCDR3 is of particular interest because it is located in the center of the Ag binding site. It contributes exceptional sequence diversity due to the variety of mechanisms that generate it, including imprecise VDJ joining, nontemplated nucleotide additions, and DH gene segment reading frame variability [24, 25]. MAb 2D6 HCDR3 is unusual both in length and content. It contains 18 a.a., framed by the conserved FR3 cysteine (Cys, a.a. 104) and FR4 tryptophan (a.a 118), compared to the average human HCDR3 length of 15.2 a.a [25]. It contains two Cys, whereas the frequency of Cys residues is typically quite low in human HCDR3: Cys constitute only 1.2–1.6% of a.a. in the HCDR3 “loop” (a.a. 107–114, site of most Ag contact) [25, 26]. Cys is underrepresented in human HC HCDR3 germline genes, compared to the genetic code, and further underrepresented in expressed HDCR3s [25]. However, Cys is naturally encoded in long human IGHD2 subgroup genes, which provide most Cys residues in human HCDR3s, and dual Cys are encoded by the IGHD2-2 allele translated in the second reading frame, as occurs in our Ag-reactive mAb. Dual cysteines allow formation of intrachain disulfide bonds that provide structural stability, particularly in long HCDR3s. Notably, HCDR3 Cys, and dual Cys in particular, are very rare in mice, in which the DH germline does not encode Cys [25, 26].

The mAb 2D6 HCDR3 Ag-binding loop sequence, VGLYCSST (a.a 107–114), also contains a rare highly hydrophobic VGLY quadrapeptide generated by somatic N-region nucleotide additions, adjacent to a IGHD2-2-encoded motif of neutral, polar Serine-Serine-Threonine a.a., in addition to Serine at residue 115, that are presumably exposed in a secondary loop formed by a Cys-Cys intrachain bond. This combination is extremely rare among human Ab. A search of the 4,752 unique human HCDR3 in the dataset of Zemlin et al. [25] found only a single human Ab HCDR3 expressing VGLY and only seven additional HCDR3, or 0.17%, using related hydrophobic motifs (VGIY, VGVY, LGVY, LGLY, LGIY, IGLY, IGVY, IGIY); none occur in Cys-containing HCDR3. The IGHD2-2-encoded CSSTSC was identified in 1.1% (50 of 4,752) of Zemlin’s dataset. The polar SSTS residues may be critical to support solubility of the adjacent hydrophobic segment. It is of note that long HCDR3s with regions of hydrophobicity have been associated with autoreactivity and polyreactivity [27, 28]; the presumptive structure of 2D6 suggests that protrusion of a long, flexible HCDR3 loop is not essential for these properties, as the Cys-Cys bond would compact the Ab binding fold.

Collectively, these results suggest that naïve B cells expressing unmutated anti-alpha3(IV)NC1 collagen Ig are present in the preimmune repertoire of some healthy individuals, including the individual that carries the genotype of the human cord blood that generated mAb 2D6. These cells are generated in part by somatically introduced residues in the HCDR3. These cells could serve as precursors in an isotype switch or affinity maturation response that yields high affinity anti-collagen IgG such as those present in patients with Goodpasture syndrome and anti-GBM glomerulonephritis. This conclusion is consistent with reports that natural anti-alpha3(IV)NC1 IgG reactive with Goodpasture epitopes can be detected in serum of healthy subjects using enrichment techniques [7]. The role of host genetic susceptibility due to predisposing germline variable gene segments is unclear, though striking interindividual variation in Ig genotypes is reported [29]. Whether these B cells would escape negative selection in a human host, as they did in the humanized mouse, is currently unknown. Binding of 2D6 to human Goodpasture antigen and pathogenic epitopes also awaits confirmation, as does the possibility of crossreactive binding to other collagen chains.

We have little evidence that immunizations induced a prominent human IgG Ag-specific response in Hu-HSC mice, echoing reports of others, including in humanized mice transplanted with human fetal thymus [30, 31]. A number of factors may prevent optimal human B and T cell interactions, and are the targets of new strategies to improve outcomes of immunization [32–34]. Use of one of these models may reveal if high affinity autoAb similar to those in Goodpasture patients are derived from a pool of natural autoAb represented by mAb 2D6. In this regard, comparison of IgM and IgG autoAb will be critical to understand origins and regulation of pathogenic Ag-specific immune responses, since IgG arise from IgM+ B cell precursors and IgM B cells are the target of initial activation by self or foreign antigen, environmental agent, mitogen, or other factor. The site at which pathogenic anti-GBM reactivity is generated (bone marrow or peripheral immune organs), the nature of tolerance defect(s) that permit the autoreactive cell survival and activation, the role of antigen crossreactivity, the role of Ab hypermutation, and the nature of activating stimuli remain unknown.

Failure to induce substantial Ag-specific human IgG may explain in part the absence of significant renal injury in immunized Hu-HSC mice, although absence of nephritis in vivo is not a reliable indicator of anti-alpha3(IV)NC1 Ab pathogenicity in this model. Immune deposition and disease induction by anti-alpha3(IV)NC1 Ab is unpredictable in mice and strain dependent. Variability in disease induction is likely due in part to genetic differences in engagement of inflammatory effector systems and to restricted in vivo exposure of pathogenic epitopes in rodent GBM due to extensive hexamer crosslinking [12]. NOD mice in particular, the strain from which NSG were derived, lack complement factor 5 and mouse IgG2a and were previously shown to develop only limited disease after alpha3(IV) collagen immunization [11]. NSG additionally carry a mutation in the IL2 receptor gamma chain that leads to defective cytokine signaling, a modification that facilitates human HSC engraftment but which may also limit inflammatory responses.

Comparison of sequences of human 2D6 mAb described here with two murine anti-alpha3(IV)NC1 collagen mAb, 1G6 and mAb3, derived from immunized mice and for which published complete sequences are available, deserves comment [35, 36]. Species-specific features are evident. Human 2D6 has fewer HCDR3 and total CDR tyrosine (Tyr) residues (16.7%, or 3 of 18 for HCDR3, and 12.7%, or 7 of 55, for all CDR) compared to 1G6 (25%, or 3 of 12, for HCDR3, and 20%, or 10 of 50, for all CDR) and to mAb3 (21%, or 3 of 14, for HCDR3, and 16.7%, or 8 of 48, for all CDR, based on IMGT CDR designations). Similar frequencies of Tyr residues that are significantly different in human vs mouse HCDR3 are reported by others [26, 27]. Cys residues are absent from HCDR3 of the mAb from mice, a species that lacks germline-encoded CDR Cys. Serine constitutes 30.9% of CDR a.a. in human mAb 2D6, compared to only 4% in murine mAb 1G6 and 10.4% in mAb3 (and 17.3% of CDR a.a. in vertebrates) [37].

These findings highlight important as yet unanswered questions regarding modeling human pathogenic immune responses in mouse. The disparate CDR a.a. composition and species-specific features suggest that the immunogen that initiates the pathogenic anti-GBM response is selecting from substantially different preimmune repertoires in mouse and man. In this regard, the mouse and human VH gene loci differ substantially [38]. Xenomouse models that replace mouse with variable subsets of human Ig genes address this issue. It is notable that a human anti-alpha3(IV)NC1 IgG2, kappa mAb, termed F1.1, derived from an immunized Xenomouse II appears to express the same IGHV4-4 gene (reported as VH4 DP-70) as does our mAb [39], and lends support to the possibility of a shared structure and idiotype among GP Ig [40]. Humanized models such as Hu-HSC that introduce a more fully humanized immune system and specifies-specific recombination machinery may be critical to correctly replicate some human immune responses. Nonetheless, if a process of convergent selection leads ultimately to similar CDR sequences or secondary and tertiary structures in the Ag binding sites through somatic processes, then study of mouse Abs will inform our understanding of their human counterparts in anti-GBM disease. In this regard, both the 2D6 and 1G6 mAb HCDR3 contain a 3′ mostly N-nucleotide-encoded hydrophobic motif (VGLY for 2D6, PPY for 1G6), suggesting that hydrophobic residues in this position may facilitate binding to alpha3(IV)NC1 collagen, and suggesting selection for this somatically-introduced motif.

In contrast to Hu-HSC mice, few EBV-transformed human B cells were recovered from Hu-PBL mice. Our rationale for using this model to sample patient cells was based in part on reports that Ag-specific B cells are quite rare in peripheral blood, such that recovery directly from this cell pool is difficult [41]. We reasoned that recovery may be enhanced if Ag-specific clones expand after transfer to lymphopenic NSG mice. In our experiments, mice injected with PBL from nephritis patient donor GP02 (n = 2) received an estimated 7x10^5^ human B cells. At spleen harvest 30 and 39 days later, human B cells comprised 5.38 and 18.20% of live cells, or 2.04–5.64 million total B cells. Yet subsequent culture with EBV failed to yield transformed cells. Our experience is consistent with that of Ifversen, who found it difficult to recover EBV-transformed B cells from humanized SCID-Hu-PBL mice [42]. Our findings further indicate that failure of EBV transformation is due in large part to loss of CD21, the EBV receptor, from B cells after transfer to NSG hosts, likely due to xenoactivation and terminal differentiation to plasma cells.

## Conclusion

Humanized models can provide critical insight into the origins of pathogenic autoantibodies in man, including origins of the elusive anti-alpha3(IV)NC1 collagen autoantibodies that destroy lungs and kidneys in patients with anti-GBM nephritis and Goodpasture syndrome. Human B cells derived from immunized Hu-HSC mice reveal that potentially nephritogenic B cells or their precursors bearing unmutated Ig receptors reactive with alpha3(IV)NC1 collagen can be generated in, and are not purged from, the human preimmune repertoire. Thus peripheral tolerance mechanisms may be critical to keep these autoimmune cells in check. The finding that uniquely human gene elements are recruited to generate the antigen binding site in at least a subset of these autoantibodies further suggests that humanized models may provide insights unobtainable from conventional mouse models.

## Additional files

Additional file 1: Figure S1. Sequences of rearranged Ig heavy chain variable region genes of human anti-alpha3(IV)NC1 collagen monoclonal antibody 2D6. Sequence of mAb 2D6 heavy chain V-D-J region and sequences of the closest corresponding germline variable region gene segments identified in the IMGT/V-QUEST reference databases, with CDR-IMGT delineated according to the IMGT unique numbering for V-DOMAIN. Additional file 2: Figure S2. Sequences of rearranged Ig light chain variable region genes of human anti-alpha3(IV)NC1 collagen monoclonal antibody 2D6. Sequence of mAb 2D6 light chain V-J region and sequences of the closest corresponding germline variable region gene segments identified in the IMGT/V-QUEST reference databases, with CDR-IMGT delineated according to the IMGT unique numbering for V-DOMAIN.
