# Supplementary material for: Recovery of a human natural antibody against the noncollagenous-1 domain of type IV collagen using humanized models
Source: J Transl Med. 2015 Jun 6;13:185. doi: 10.1186/s12967-015-0539-4 (PMC4467618; doi:10.1186/s12967-015-0539-4)
Supplement: Additional file 1: Figure S1. — Sequences of rearranged Ig heavy chain variable region genes of human anti-alpha3(IV)NC1 collagen monoclonal antibody 2D6. Sequence of mAb 2D6 heavy chain V-D-J region and sequences of the closest corresponding germline variable region gene segments identified in the IMGT/V-QUEST reference databases, with CDR-IMGT delineated according to the IMGT unique numbering for V-DOMAIN. [file 12967_2015_539_MOESM1_ESM.pdf]

Additional file 1

```
                        <---------------------------------------------  FR1 - IMGT
                         1              5                   10                  15
                         Q   V   Q   L   Q   E   S   G   P       G   L   V   K   P
2D6_heavy_chain         cag gtg cag ctg cag gag tcg ggc cca ... gga ctg gtg aag cct
IGHV4-4*02              --- --- --- --- --- --- --- --- --- ... --- --- --- --- ---

                        ------------------------------------------->  ________________
                                        20                  25                  30
                         S   G   T   L   S   L   T   C   A   V   S   G   G   S   I
2D6_heavy_chain         tcg ggg acc ctg tcc ctc acc tgc gct gtc tct ggt ggc tcc atc
IGHV4-4*02              --- --- --- --- --- --- --- --- --- --- --- --- --- --- ---

                        __  CDR1 - IMGT  ______________ <-------------------------
                                        35                  40                  45
                         S               S   S   N   W   W   S   W   V   R   Q   P
2D6_heavy_chain_        agc ... ... ... agt agt aac tgg tgg agt tgg gtc cgc cag ccc
IGHV4-4*02              --- ... ... ... --- --- --- --- --- --- --- --- --- --- ---

                        FR2 - IMGT  ------------------------->  ______________  CDR2
                                        50                  55                  60
                         P   G   K   G   L   E   W   I   G   E   I   Y   H   S
2D6_heavy_chain         cca ggg aag ggg ctg gag tgg att ggg gaa atc tat cat agt ...
IGHV4-4*02              --- --- --- --- --- --- --- --- --- --- --- --- --- --- ...

                        - IMGT  __________ <---------------------------------------
                                        65                  70                  75
                                 G   S   T   N   Y   N   P   S   L   K       S   R
2D6_heavy_chain         ... ... ggg agc acc aac tac aac ccg tcc ctc aag ... agt cga
IGHV4-4*02              ... ... --- --- --- --- --- --- --- --- --- --- ... --- ---

                        -------------------------------  FR3 - IMGT  --------------
                                        80                  85                  90
                         V   T   I   S   V   D   K   S   K   N   Q   F   S   L   K
2D6_heavy_chain         gtc acc ata tca gta gac aag tcc aag aac cag ttc tcc ctg aag
IGHV4-4*02              --- --- --- --- --- --- --- --- --- --- --- --- --- --- ---

                        ----------------------------------------------------->  ____
                                        95                  100             104
                         L   S   S   V   T   A   A   D   T   A   V   Y   Y   C   A
2D6_heavy_chain         ctg agc tct gtg acc gcc gcg gac acg gcc gtg tat tac tgt gcg
IGHV4-4*02              --- --- --- --- --- --- --- --- --- --- --- --- --- --- ---

                        __________________________  CDR3 - IMGT  __________________
                         R   V   G   L   Y   C   S   S   T   S   C   Y   Y   G   M
2D6_heavy_chain         aga gta ggg cta tat tgt agt agt acc agc tgc tac tac ggt atg
IGHV4-4*02              --- -
N-region nucleotides         nn nnn nn
IGHD2-2*01                             - --- --- --- --- --- --- --
IGHJ6*02                                                          - --- --- --- ---

                        _______
                         D   V   W   G   Q   G   T   T   V   T   V   S   S
2D6_heavy_chain         gac gtc tgg ggc caa ggg acc acg gtc acc gtc tcc tca
IGHJ6*02                --- --- --- --- --- --- --- --- --- --- --- --- ---
```

Additional File 1 Figure Legend. Sequences of rearranged Ig heavy chain variable region genes of human anti-alpha3(IV)NC1 collagen monoclonal antibody 2D6. The mAb was derived from an immunized Hu-HSC mouse. Dashes represent sequence identity with 2D6. Dots represent empty spaces introduced to maximize sequence homologies. The sequences of the closest corresponding germline V region gene segment alleles identified in the IMGT/V-QUEST reference database are shown. CDR-IMGT are delineated according to the IMGT unique numbering for V-DOMAIN [1]. The heavy chain CDR-IMGT lengths (amino acids) are 9.7.18.

Reference:

1. Lefranc MP, Pommie C, Ruiz M, Giudicelli V, Foulquier E, Truong L, et al: **IMGT unique numbering for immunoglobulin and T cell receptor variable domains and Ig superfamily V-like domains**. *Dev Comp Immunol* 2003, **27**(1):55-77.
