# Supplementary material for: Recovery of a human natural antibody against the noncollagenous-1 domain of type IV collagen using humanized models
Source: J Transl Med. 2015 Jun 6;13:185. doi: 10.1186/s12967-015-0539-4 (PMC4467618; doi:10.1186/s12967-015-0539-4)
Supplement: Additional file 2: Figure S2. — Sequences of rearranged Ig light chain variable region genes of human anti-alpha3(IV)NC1 collagen monoclonal antibody 2D6. Sequence of mAb 2D6 light chain V-J region and sequences of the closest corresponding germline variable region gene segments identified in the IMGT/V-QUEST reference databases, with CDR-IMGT delineated according to the IMGT unique numbering for V-DOMAIN. [file 12967_2015_539_MOESM2_ESM.pdf]

Additional file 2

```
                                   <--------------------------------------------  FR1 — IMGT
                                   1               5                   10                  15
                                    Q   S   A   L   T   Q   P   A   S       V   S   G   S   P
2D6_lambda_light_chain             cag tct gcc ctg act cag cct gcc tcc ... gtg tct ggg tct cct
IGLV2-14*03                        ... ... ... ... ... ... ... ..- --- ... --- --- --- --- ---

                                   ------------------------------------------> ________________
                                                   20                  25                  30
                                    G   Q   S   I   T   I   S   C   T   G   T   S   S   D   V
2D6_lambda_light_chain             gga cag tcg atc acc atc tcc tgc act gga acc agc agt gac gtt
IGLV2-14*03                        --- --- --- --- --- --- --- --- --- --- --- --- --- --- ---

                                   __  CDR1 - IMGT  ______________ <-------------------------
                                                    35                  40                  45
                                    G               G   Y   N   Y   V   S   W   Y   Q   Q   H
2D6_lambda_light_chain             ggt ... ... ... ggt tat aac tat gtc tcc tgg tac caa caa cac
IGLV2-14*03                        --- ... ... ... --- --- --- --- --- --- --- --- --- --- ---

                                   FR2 - IMGT  -------------------------> ______________  CDR2
                                                  50                  55                  60
                                    P   G   K   A   P   K   L   M   I   Y   D   V
2D6_lambda_light_chain             cca ggc aaa gcc ccc aaa ctc atg att tat gat gtc ... ... ...
IGLV2-14*03                        --- --- --- --- --- --- --- --- --- --- --- --- ... ... ...

                                   - IMGT  __________ <----------------------------------------
                                                  65                  70                  75
                                                    S   N   R   P   S   G   V   S       N   R
2D6_lambda_light_chain              ... ... ... ... agt aat cgg ccc tca ggg gtt tct ... aat cgc
IGLV2-14*03                         ... ... ... ... --- --- --- --- --- --- --- --- ... --- ---

                                    -------------------------------  FR3 - IMGT  ---------------
                                                    80                  85                  90
                                     F   S   G   S   K           S   G   N   T   A   S   L   T
2D6_lambda_light_chain              ttc tct ggc tcc aag ... ... tct ggc aac acg gcc tcc ctg acc
IGLV2-14*03                         --- --- --- --- --- ... ... --- --- --- --- --- --- --- ---

                                    -----------------------------------------------------> ____
                                                    95                  100             104
                                     I   S   G   L   Q   A   E   D   E   A   D   Y   Y   C   S
2D6_lambda_light_chain              atc tct ggg ctc cag gct gag gac gag gct gat tat tac tgc agc
IGLV2-14*03                         --- --- --- --- --- --- --- --- --- --- --- --- --- --- ---

                                    _______  CDR3 -  IMGT _________
                                     S   Y   T   S   S   S   T   V   F   G   G   G   T   K   L
2D6_lambda_light_chain              tca tat aca agc agc agc act gta ttc ggc gga ggg acc aag ctg
IGLV2-14*03 F                       --- --- --- --- --- --- ---
IGLJ2*01/IGLJ3*01                                               --- --- --- --- --- --- --- ---

                                     T   V   L   G   Q   P   K   A   A   P   S   V   T   L   F
2D6_lambda_light_chain              acc gtc cta ggt cag ccc aag gct gcc ccc tcg gtc act ctg ttc
IGLJ2*01/IGLJ3*01                   --- --- --- -
Human lambda constant region:                     -- --- --- --- --- --- --- --- --- --- --- ---
```

Additional File 2 Figure Legend. Sequences of rearranged Ig light chain variable region genes of human anti-alpha3(IV)NC1 collagen monoclonal antibody 2D6. 2D6 is derived from an immunized Hu-HSC mouse. Dashes represent sequence identity with 2D6. Dots represent empty spaces introduced to maximize sequence homologies, except at the most 5' end of IGLV2-14*03, where they reflect the first 8 codons of FR1 of this allele which are missing in the current databases. The sequences of the closest corresponding germline V region gene segment alleles identified in the IMGT/V-QUEST reference database are shown. CDR-IMGT are delineated according to the IMGT unique numbering for V-DOMAIN [1]. CDR-IMGT lengths (amino acids) are 9.3.9 for the light chain.

Reference:

1. Lefranc MP, Pommie C, Ruiz M, Giudicelli V, Foulquier E, Truong L, et al: **IMGT unique numbering for immunoglobulin and T cell receptor variable domains and Ig superfamily V-like domains**. *Dev Comp Immunol* 2003, **27**(1):55-77.
